# Assessment of the Relevance and Reliability of Reaction Time Tests Performed in Immersive Virtual Reality by Mixed Martial Arts Fighters

**DOI:** 10.3390/s22134762

**Published:** 2022-06-24

**Authors:** Jacek Polechoński, Alan Langer

**Affiliations:** 1 Institute of Sport Sciences, The Jerzy Kukuczka Academy of Physical Education in Katowice, 40-065 Katowice, Poland; 2 Student Scientific Circle of Physical Activity and Tourism in Virtual Reality “ACTIVE VR” The Jerzy Kukuczka Academy of Physical Education in Katowice, 40-065 Katowice, Poland; langermma@gmail.com

**Keywords:** reaction time, immersive virtual reality, VR, mixed martial arts, MMA, martial arts

## Abstract

Immersive virtual reality (VR) is increasingly applied in various areas of life. The potential of this technology has also been noticed in recreational physical activity and sports. It appears that a virtual environment can also be used in diagnosing certain psychomotor abilities. The main aim of this study consisted of assessing the relevance and reliability of VR-implemented tests of simple and complex reaction time (RT) performed by mixed martial arts (MMA) fighters. Thirty-two professional MMA fighters were tested. The original test developed in the virtual environment was applied for RT assessment. The fighters’ task consisted of reacting to the lighting up of a virtual disc situated in front of them by pushing a controller button. The relevance of the test task was estimated by juxtaposing the obtained results with the classic computer test used for measuring simple and complex reactions, while its reliability was assessed with the intraclass correlation procedure. Significant relationships found between the results of VR-implemented tests and computer-based tests confirmed the relevance of the new tool for the assessment of simple and complex RT. In the context of their reliability, RT tests in VR do not differ from tests conducted with the use of standard computer-based tools. VR technology enables the creation of tools that are useful in diagnosing psychomotor abilities. Reaction time tests performed by MMA fighters with the use of VR can be considered relevant, and their reliability is similar to the reliability obtained in computer-based tests.

## 1. Introduction

The notion of immersive virtual reality (VR) refers to an environment artificially created with the use of information technology, in which a human is cut off from visual and auditory stimuli from the real environment and instead proceeds with the image and sound, or even tactile sensations, of the simulated world [1,2]. VR uses customized and advanced software and hardware to create a digital 3D reality in which all user’s senses are stimulated with computer-generated sensations and feedback [3]. Therefore, VR allows participants to experience simulated digital realities similar to those of physical reality, thus creating scenarios that are impossible to experience in the real world [4]. This newly created technology is increasingly applied in various areas of life, such as industry [5], medicine, rehabilitation and healthcare [6,7,8,9,10,11] or education [12,13]. The potential of this technology has also recently been noticed in the context of health-promoting physical activity [14,15,16,17,18], its application for physical education [19], cognitive functions training [20,21], as well as various applications in sport, among which virtual training and performance analysis are the most frequently quoted [22,23].

As proved by the research performed within the last few years, the virtual environment is suitable for exercises aimed at improving fitness and performance [24,25]. It is possible thanks to appropriate applications and active video games (AVGs). Some of them are compatible with training devices such as multi-directional treadmills, cycloergometers, rowing simulators, etc. It was proven that locomotive movements promote enhanced physical effort, increase immersion and receive positive user assessment [16,17,26,27,28,29]. In connection with this fact, research is beginning to be undertaken in connection with the analysis of movement in VR [30,31,32]. It has even been observed that physical effort with the use of training devices in VR can be more intense than similar physical activity during conventional training sessions [27].

Moreover, it appears that modern technology can be useful for developing precise and objective tools for diagnosing fighters’ exercise capacity and motor potential. VR can be freely modeled, and user peripheral devices (goggles, controllers, haptic gloves and suits) are equipped with numerous advanced motion sensors that, combined with a computer and appropriate software, make it possible to analyze human body movements in real-time. The precise assessment of motion parameters can be applied in the difficult-to-diagnose sphere of coordination motor abilities. A particularly problematic issue is constituted by the analysis of movement activities connected with fast decision-making due to the necessity to perform the measurements in very short time intervals. For this reason, for the assessment of the speed of reaction to stimuli, computer technology has been applied for several dozen years [33]. All indications are that in comparison to standard computer tests, the opportunities ensured by VR are much wider. This makes it possible to create the environment imitating the fighter’s real training conditions, and the tests can be performed in various positions and even during movement. What is more, one of the significant advantages of VR consists of the possibility to control various parameters of users’ sensations in the virtual world. Distractors are absent in VR as well; the user is cut off from external stimuli that could interfere with the course of the test.

Nevertheless, in the context of developing and conducting psychomotor tests, the virtual environment has not been analyzed to a sufficient extent yet. It remains unknown whether the users will react in a similar way to stimuli of different kinds (auditory, visual, tactile) in VR and the real environment; remaining for a longer period of time in the world isolated from external signals will be considered comfortable and will not affect test results. Another aspect that needs to be taken into consideration refers to whether the results of measurements performed in a fully immersive virtual environment will correlate with the results of tests carried out based on traditional solutions. The first attempts undertaken by Vahle et al. [34] aimed at comparing the results of cognitive performance tests in VR and real-life (RL) brought very promising conclusions. According to the authors, the use of lifelike VR environments for cognitive performance tests seems not to lead to any performance changes compared to RL computer-based assessments, making VR suitable for similar applications.

As not enough scientific papers connected with this topic are available, further VR analyses need to be performed in the context of the possibility of using this technology for developing and conducting the tests relating to motor abilities. First, attempts to evaluate simple human reactions in VR need to be made, followed by the analysis of more complex cognitive processes. The basic indicator of the ability to react, process and respond to external stimuli is constituted by reaction time (RT), which is measured by the elapsed time between stimulus onset and an individual’s response to elementary cognitive tasks (ECTs), which are relatively simple perceptual-motor tasks typically administered in a laboratory setting [35]. Although ECTs may be cognitively simple, there is evidence that performance on such tasks correlates well with other measures of general intelligence [36]. Reaction time is a good indicator of sensorimotor coordination and performance of an individual; it also determines the alertness of a person and must be lesser in certain occupations, for example, drivers, military people, pilots and security guards, where alertness is a must for them [37]. For similar reasons, an appropriate RT level is of key significance in sports [38].

In numerous sports disciplines, RT constitutes a decisive factor in determining success. Fast reaction to stimuli plays a crucial role in martial arts [39]. During a fight, sportsmen constantly react to one another. Each swing, punch and step taken by the opponent requires a fast reaction. For this reason, in order to fight, it is necessary to remain constantly focused on the opponent’s movements and make rapid decisions depending on the circumstances. A slight delay in response may result in losing the fight, while reaching a higher RT level should bring higher efficiency of the applied techniques. It is necessary to emphasize that in martial arts, greater importance is attributed to visual than auditory stimuli. Mixed martial arts (MMA) constitute a discipline that combines different fighting styles. This sport is very attractive to spectators, and it has gained wide popularity [40].

Our study includes the assessment of the relevance and reliability of original RT tests developed in VR and based on the measurements performed within a group of MMA fighters, considered representative of various martial arts. What is more, an attempt has been made to answer the question of whether the applied test type (VR tests vs. computer-based tests) differentiates the RT of MMA fighters and if there exists a difference in RT for the left hand and right hand of fighters subject to tests. According to our knowledge, the study we conducted constitutes one of the first attempts to assess the possibility of using VR for developing and performing the tests aimed at measuring the reaction time of martial arts fighters.

## 2. Materials and Methods

Thirty-two professional MMA fighters were subject to tests (age 26.4 ± 5.5 years, body height 178.6 ± 5.8 cm, body weight 76.4 ± 9.8 kg, training experience 7.0 ± 4.2 years) and training in several sports clubs in southern Poland. The majority of study subjects (28 sportsmen) were right-handed, while the remaining 4 of them were left-handed. The measurements were performed at the MMA & Performance Training Studio (Świętochłowice, Poland). Study participants had to meet the following inclusion criteria: good overall health condition, no contraindications to take part in the study (in particular, no history of motion sickness, epileptic episodes, sensitivity to flashing light), no physical limitations (e.g., injuries), not performing intense physical exercise within the period of 12 h before the test and not taking medicaments that could affect reaction time. All fighters were informed about the objective and course of the tests. The study was conducted according to the guidelines of the Declaration of Helsinki and reviewed and approved by the Research Ethics Committee of the Jerzy Kukuczka Academy of Physical Education in Katowice. Informed consent was obtained from all subjects involved in the study. All participants took part in the study voluntarily and could discontinue their participation at any time. The participants had some experience connected with VR in the past, but none of them declared their use of this technology on a regular basis. They had not used the software used in RT assessment tests before either. An autonomous VR headset, Oculus Quest 2 (Facebook Technologies, LLC. 1 Hacker Way, Menlo Park, CA 94025, USA), was used for VR projection during the tests, consisting of a head-mounted display (HMD) and controllers.

**Figure 1:**
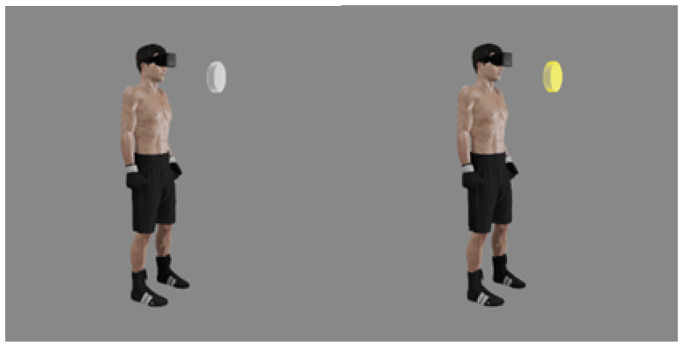
Visualization of the test used for analyzing simple reactions in VR (gray—waiting for the stimulus, yellow—right hand reaction).

**Figure 2:**
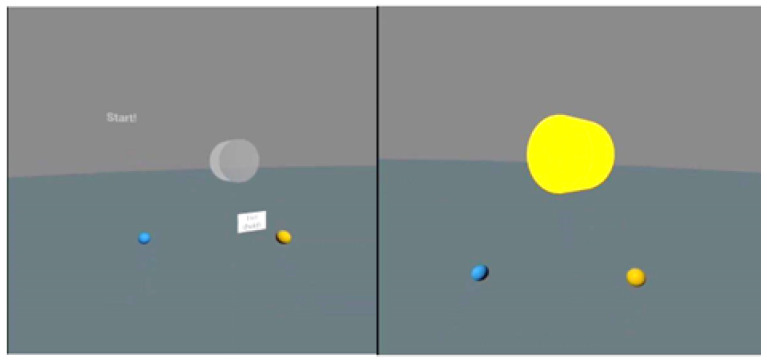
User view of the test—simple reaction in VR (gray—waiting for the stimulus, yellow—right hand reaction).

**Figure 3:**
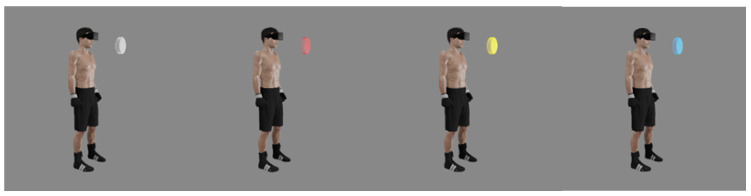
Visualization of the test used for analyzing complex reaction in VR (gray—waiting for the stimulus, red—no reaction, yellow—right hand reaction, blue—left hand reaction).

**Figure 4:**
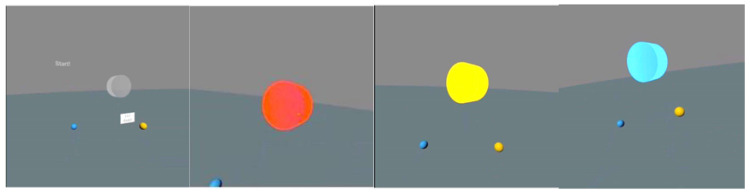
User view of the test—complex reaction in VR (gray—waiting for the stimulus, red—no reaction, yellow—right hand reaction, blue—left hand reaction).

Simple and complex RT was assessed in VR with the use of the authors’ original tests. Before the tests began, study subjects were given instructions connected to controlling VR projection devices and using the application. The fighters’ task consisted of reacting to the lighting up of the virtual disc (diameter 20 cm) situated in front of them at a distance of 30 cm by pressing the controller button. The tests were conducted in a relaxed standing position, with the feet hip-width apart and arms along the body. The tested hand held the controller in a way for the index finger to remain on its dedicated button (trigger). The task of the study participant consisted of pressing the controller button when a lighting stimulus (lighting up of the disc) appeared (Figure 1 and Figure 2). A simple reaction test was performed for the right hand and left hand separately. Three different responses to the signal were possible during the assessment of complex reactions. When the disc lit up in yellow, the user pressed the button of the controller in his right hand with his right index finger, while when the light was blue, he pressed the button of the controller in his left hand with his left index finger, while the study subject was supposed to not react to the red color (Figure 3 and Figure 4). The signal appeared on a randomized basis at intervals from 2–6 s. The stimulus type was also randomly generated, while each signal type was presented an equal number of times. The next stimulus was generated after the user’s response to the previous signal. RT was calculated during the test, referring to the time that elapsed from the moment of activating a visual stimulus to the moment of the user pressing the controller button. The software recorded subsequent reaction times in milliseconds (ms). The fighters performed a mock test consisting of 6 stimuli. The next step consisted of three separate proper tests, including 12 impulses with their results recorded. The average reaction time was calculated for each test, excluding two extreme values. Average results calculated in this way were used for assessing test reliability.

**Figure 5:**
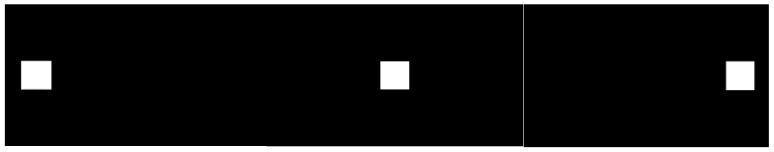
Visualization of visual stimuli appearing on the screen during computer-based tests intended for measuring the time of simple and complex reactions.

In order to determine the relevance of the new VR-based RT test, similar measurements were performed with the use of classic computer-based tests [41]. They consisted of pressing appropriate keys on a computer keyboard when light stimuli (white squares) appeared on the screen. The research procedure was the same as the procedure applied for the tests in VR. A simple reaction test was conducted for the right hand and the left hand separately. Study subjects pressed a defined key on the keyboard with their index finger when a white square appeared in the middle of the screen. When it comes to complex reaction tests, white squares could appear in three places (left side, right side or the middle of the screen). Study subjects pressed the marked keys accordingly: with their left or right index finger when the square appeared on the sides of the screen or space with any thumb when it appeared in the middle (Figure 5).

The relevance of test tasks developed in the virtual environment was assessed with the use of Pearson’s correlation analysis. The results obtained in VR were juxtaposed with the results of classic computer-based tests. Intraclass correlation (ICC) procedures were applied for the purpose of evaluating measurement reliability [42,43]. Two-factor mixed-effects model, in which subject effects are random, while position effects fixed were applied together with intraclass correlation type relying on the definition of absolute capacity. Reliability was assessed for a single measurement. The F test was applied to check the reliability of the intraclass correlation coefficient. Reliability coefficients at or greater than 0.90 were considered very high, 0.80–0.89 were considered high, 0.70–0.79 were considered adequate, 0.60–0.69 were marginal and 0.59 or lowerwere considered low [44,45]. Comparison of the reaction of the right hand and left hand was also performed, and reaction times in VR and outside VR were juxtaposed. Normality was evaluated with the use of the Shapiro–Wilk test. The significance of differences in the obtained results was estimated with the use of variance analysis and NIR post hoc tests (simple RT), and the Student t-test (complex RT). The study admitted the value of *p* < 0.05 for statistical significance. Statistical calculations were performed using IBM SPSS Statistics and Statistica software (StatSoft, Inc., Tulsa, OK, USA).

## 3. Results

**Figure 6:**
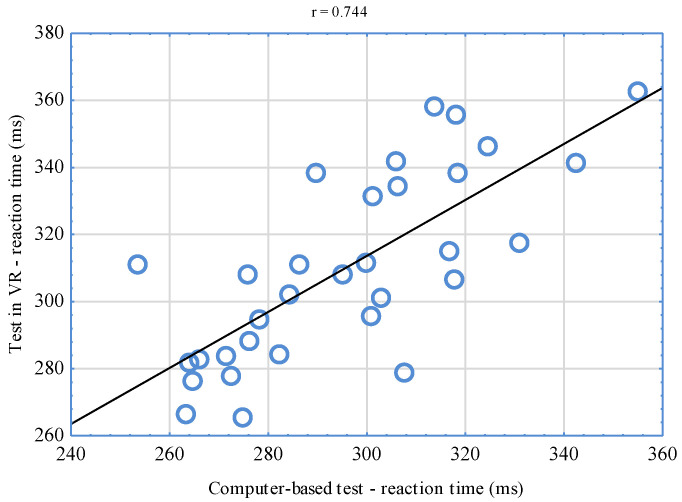
Correlation of the results of simple reaction tests in VR and computer-based ones—right hand.

**Figure 7:**
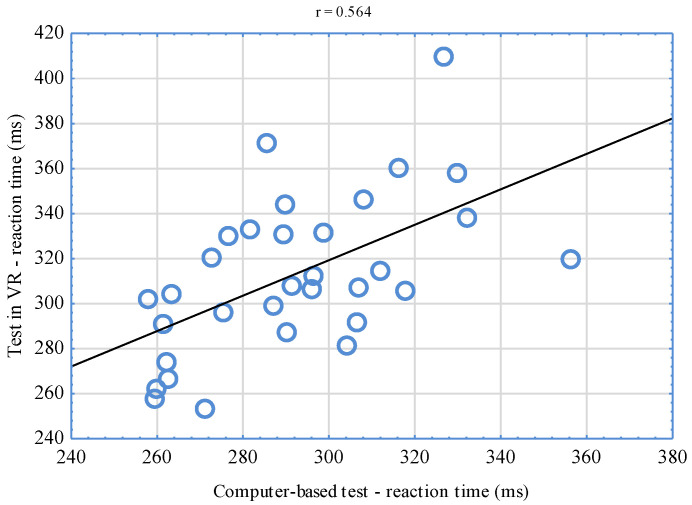
Correlation of the results of simple reaction tests in VR and computer-based ones—left hand.

**Figure 8:**
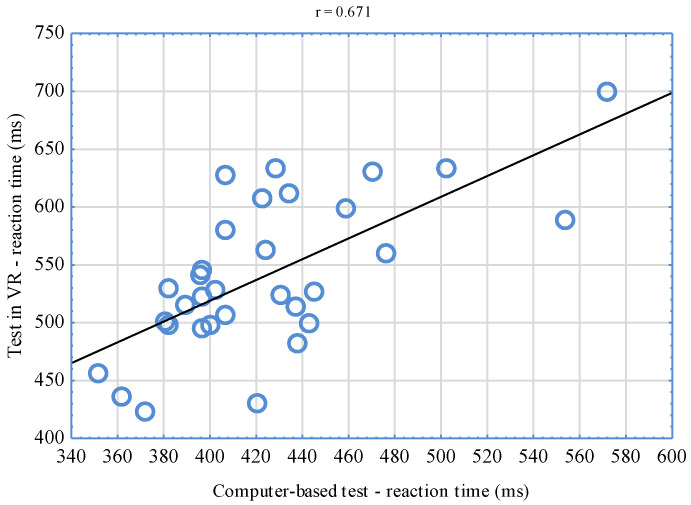
Correlation of the results of complex reaction tests in VR and computer-based ones.

The conducted analyses of the correlation between VR-implemented simple RT tests and computer-based tests indicate significant relationships (*p* < 0.001). The correlation coefficient reached the level of r = 0.744 for the right hand (Figure 6) and r = 0.564 for the left hand (Figure 7). Having juxtaposed the results of tests assessing complex reactions, a significant relationship (*p* < 0.001) was also stated. In this case, the correlation coefficient was r = 0.671 (Figure 8).

Intraclass correlation coefficients ICC for VR-implemented reaction tests and computer-based tests reached a similar level. All correlations were statistically significant (*p* < 0.001). In the case of computer-based simple RT tests of the right hand, a slightly lower value of intraclass correlation coefficient was observed (ICC = 0.793) compared to the left hand (ICC = 0.836). For complex reactions, the ICC value was 0.743 (Table 1). For tests developed in the virtual environment, the highest value of intraclass correlation coefficient was recorded for the simple reaction test of the left hand (ICC = 0.805), slightly lower than for the right hand (ICC = 0.730), while for complex reaction test ICC reached 0.801 (Table 2).

**Figure 9:**
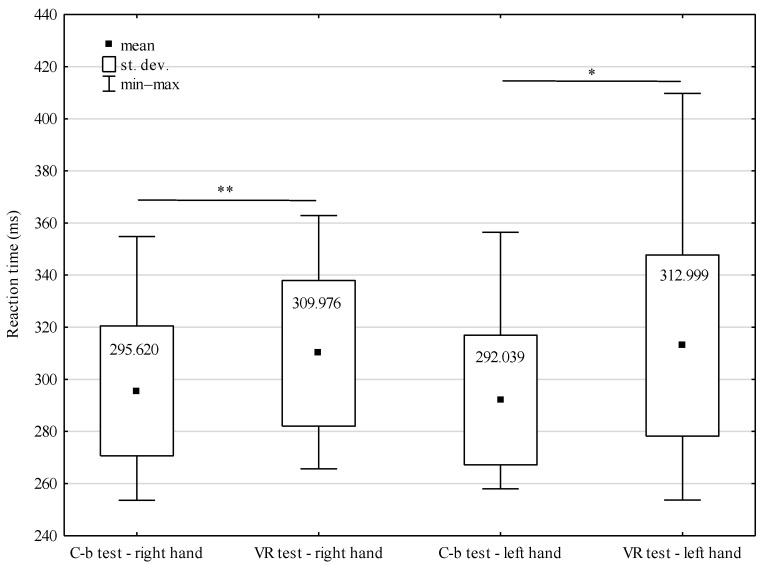
Simple RT of MMA fighters during the tests in VR and computer-based (C-b) tests, * *p* < 0.05, ** *p* < 0.01.

**Figure 10:**
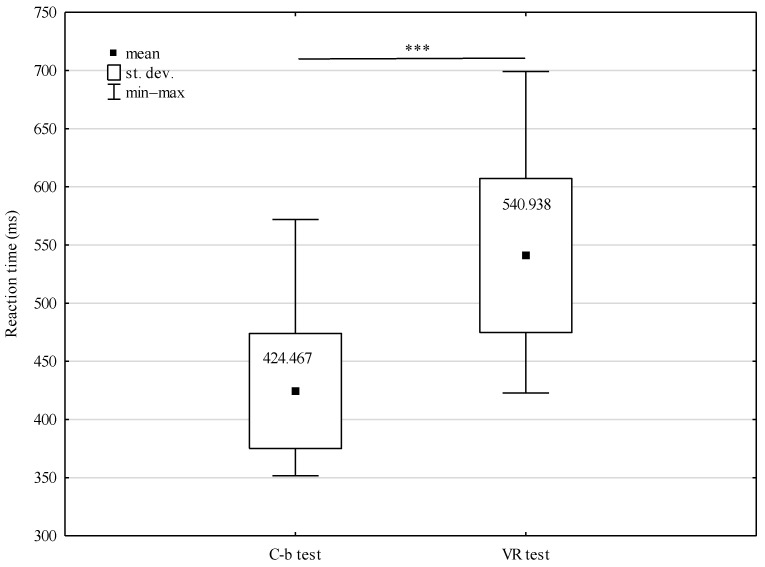
Complex RT of MMA fighters during the tests in VR and computer-based (C-b) tests, *** *p* < 0.001.

The conducted analysis of variance shows a significant influence of the type of tests performed on simple RT results (F = 6.792; *p* < 0.001). Statistically significant differences were manifested after juxtaposing simple reaction times in VR and during computer-based tests. In the case of the left hand, the difference was 20.960 ms (*p* < 0.05), while for the right hand, it reached the level of 14.356 ms (*p* < 0.01). In turn, it was not significant for RT whether the tests were performed using the right hand or the left hand (F = 0.017; *p* = 0.896). In computer-based tests, the difference in reaction times amounted to only 3.581 ms in favor of the left hand, while in the tests conducted in a virtual environment, it was 3.023 ms in favor of the right hand (Figure 9). Additionally, complex reaction times in VR and during computer-based tests differed significantly with 116.471 ms (*p* < 0.001) (Figure 10).

## 4. Discussion

The significant relationships found between the results of VR-implemented tests and computer-based tests confirm the relevance of the new tool for the assessment of simple and complex RT. Based on the classification suggested by Mukaka [46], when it comes to the right hand, the value of the correlation coefficient of the results of computer-based tests and the tests conducted in VR was high (r = 0.744). Moderate interdependencies were, in turn, observed for tests performed with the left hand (r = 0.564) as well as the right and left hand (r = 0.671)—complex reaction. It is difficult to unequivocally interpret higher coefficient values for the right hand. It could have been influenced by laterality. It is necessary to mention that a decisive majority of sportsmen (87.5%) who took part in the study were right-handed. It is also possible to assume that correlations of the obtained results could have reached even higher levels but for certain differences between the compared tests. It should be emphasized that the tests differed from one another not only when it comes to the environment in which they were conducted but also with their graphic layer. Differences consisted in the fact that in VR, MMA fighters reacted to changing colors of virtual discs, while in computer-based tests, they reacted to white squares appearing on a black background. It is difficult to refer the results obtained in own research to the findings of other authors, as there are no counterparts. Similar research experiments were conducted by Vahle et al. [34], who compared the results of cognitive performance tests based on reaction time in VR and RL. For the purposes of their experiments, the authors developed a virtual environment that was a copy of an RL laboratory hall. In both study locations, the subjects performed the same tests. Their results were compared, but no significant differences were observed. According to the authors, the use of lifelike VR environments for cognitive performance tests seems not to lead to any performance changes compared to RL computer-based assessments, making VR suitable for similar applications.

In the context of their reliability, RT tests in VR do not differ from tests conducted with the use of standard computer-based tools. In VR, test results for RT of the left hand and complex reactions fulfilled the criteria of high reliability (0.80 ≤ ICC ≤ 0.89), while for the right hand, they were of adequate reliability (0.70 ≤ ICC ≤ 0.79). In classic computer-based tests, the reliability of RT results for the left hand was high (0.80 ≤ ICC ≤ 0.89), while for the right hand and complex reaction, it was at an adequate level (0.70 ≤ ICC ≤ 0.79). The values of reliability coefficients of simple and complex RT obtained in our own studies are comparable and in some cases, higher with reference to the results previously reported by other authors using computerized cognitive assessment tools (CCATs). Cole et al. [44], while performing a neuropsychological assessment of soldiers with the use of CCATs, established that the results of ICC of RT tests used in their studies reach the level of 40–75 ICC. Similar results were also obtained by Farnsworth et al. [47] in their study group consisting of young sportspeople. The researchers assessed the reliability of four cognitive tasks forming part of CCATs. The reliability between repeated measurements of the conducted tests was at a low or marginal level of significance (ICC between 0.401–0.672). Interesting studies of reaction time with the participation of military staff were performed by Soares et al. [48]. The authors developed the software, so it was compatible with a controller in the shape of a gun. Study subjects performed two types of tasks. The first test assessed simple reactions and consisted in pressing the trigger when the image of a criminal appeared on the screen. The second test was aimed at assessing choice RT. Two buttons were used for this purpose (trigger and button under the trigger). The task of a study participant consisted in reacting to the appearing images. When the image of a criminal was displayed, they were supposed to press the trigger, and when the image of a victim appeared, the task consisted of pressing the button below. The authors considered the ICC ≥ 0.70 as satisfactory. Depending on their age, test participants achieved ICC for simple reaction within the range 0.808–0.821, while for choice RT, their results were within the range of 0.802–0.868. However, the presented comparisons should be treated as approximate, as it is necessary to emphasize that the quoted authors conducted their tests and analyses following different procedures.

The study also included the comparison of the results of VR-based and computer-based tests. It was concluded that both for simple as well as complex reactions, the fighters achieved significantly better results outside the virtual environment. It is difficult to unequivocally determine the cause of such dependencies. One of the reasons may be the delay caused by hardware or software [33]. It is also necessary to emphasize that, in the case of computer-based tests, the fighters pressed keys on the keyboard wired to the computer, while controllers used during the tests in VR were connected with autonomous HMD Oculus Quest 2 wirelessly. The application of wireless connection could extend signal transmission, thus resulting in longer reaction times being recorded in VR. Achieving significantly better results during computer-based tests can also be connected with the fact that study participants were not accustomed to the virtual environment. All fighters declared to have some experience with VR, but they never used this technology regularly beforehand. It is necessary to remember that the speed of our cognitive and perceptive processing is directly connected with the complexity of stimuli [49]. It is thus probable that the combination of cognitive tasks with a new element and multisensory VR environment could result in the overload of the nervous system and thus in longer perception time and time of reaction to stimuli. Nevertheless, such an assumption is questioned by the research conducted previously by Vahle et al. [34], who compared the results of identical cognitive performance tests based on RT in VR and RL, and no significant differences were reported between them.

The study compared the results of tests performed with the right hand and left hand in VR and outside this technology, but irrespective of the environment in which MMA fighters were subject to tests, no statistically significant differences in RT were observed between the tested hands. It is, however, necessary to notice that in VR, slightly shorter RT was observed for the right hand while outside VR for the left hand. Badau et al. [50] performed three types of RT tests at various levels of complexity among 332 sportspeople representing different individual sports (boxing, gymnastics, judo, karate, taekwondo, wrestling). Research results show that in the case of a simple test task, better results were achieved by sportspeople for their left hand, while the more complex the test, the better performance was recorded for their right-dominating hand. This fact can be connected with lateralization and different specializations of both brain hemispheres, representing the right and left arm [51]. As far as our research is concerned, the test conducted in VR should be treated as more complex due to its multisensory environment, where similarly to the abovementioned study, better results were observed for the left hand. Nevertheless, it is necessary to emphasize that in the context of the domination of the right or left arm with reference to RT, research results presented by other authors are not conclusive. Some of them show that simple RT for the left hand is shorter than for the right hand [52,53,54,55], while the others present a reverse dependency [56,57].

## 5. Limitations

There is no doubt that the results presented in the study have several limitations. The relatively small number of study participants makes it necessary to adopt a careful approach to the obtained results. In the context of relevance assessment, it is necessary to emphasize that the compared tests were not identical. They were set not only in different environments, but also their graphic layer differed. It is recommended to compare more similar tests in the future. The difference should refer exclusively to the testing environment. It also seems justified to determine equipment delay time, which was not taken into consideration in the conducted study.

## 6. Conclusions

To sum up, it is necessary to emphasize that the tests presented in the study constitute one of the first research tools for RT assessment developed with the use of VR technology. Their relevance and reliability, similar to computer-based tests, prove the necessity to adopt a serious approach towards the idea of creating applications of this kind for the purposes of sports tests and maybe also psychological and medical diagnostics. Due to the innovative character of the conducted study, it is necessary to organize further scientific experiments of a similar character in order to confirm the potential of the scientific environment in the context of performing the measurements of cognitive abilities and searching for further directions for the development and application of this advanced technology. The next stage may consist in creating objective motor skills tests in VR based on specific motion patterns directed at concrete sports disciplines and even establishing training programs. It should be emphasized that the study presented in the paper forms part of a larger scientific project aimed at creating tests aimed at diagnosing the level of coordination motor skills and teaching boxing skills. The choice of such a research topic was motivated by the high significance of coordination performance in fight sports [58,59,60,61,62,63].

Even if, until recently, exercises in the virtual environment were used mainly by beginner sportspeople [64], it seems that they will become increasingly popular in professional sports, as VR-based training may be really useful for them. In a virtual environment, it seems possible, for example, to practice dangerous skills without the risk of injury. In turn, when it comes to the advantages of developing motor tests in VR, it is possible to enumerate, among others: the possibility to conduct the test on their own by a sportsperson, measurement objectivity, the possibility of submitting the results to the trainer for fast analysis and the absence of distractors, e.g., the user is cut off external stimuli that may have a negative effect on the course of the test.

## Figures and Tables

**Table 1:** Intraclass correlation coefficients (ICC) for computer-based simple and complex reaction tests.

Type of Test	ICC	*p*
Simple reaction—right hand	0.793	0.001
Simple reaction—left hand	0.836	0.001
Complex reaction—right and left hand	0.743	0.001

**Table 2:** Intraclass correlation coefficients (ICC) for VR-implemented simple and complex reaction tests.

Type of Test	ICC	*p*
Simple reaction—right hand	0.730	0.001
Simple reaction—left hand	0.805	0.001
Complex reaction—right and left hand	0.801	0.001

## Data Availability

The data presented in this study are available on request from the corresponding author.
